# Significant THz absorption in CH_3_NH_2_ molecular defect-incorporated organic-inorganic hybrid perovskite thin film

**DOI:** 10.1038/s41598-019-42359-8

**Published:** 2019-04-09

**Authors:** Inhee Maeng, Young Mi Lee, Jinwoo Park, Sonia R. Raga, Chul Kang, Chul-Sik Kee, Byung Deok Yu, Suklyun Hong, Luis K. Ono, Yabing Qi, Min-Cherl Jung, Masakazu Nakamura

**Affiliations:** 1 Advanced Photonics Research Institute, Gwangju Institute of Science and Technology, Gwangju, 61005 Republic of Korea; 2 Beamline department, Pohang Accelerator Laboratory, POSTECH, Pohang, 37673 Republic of Korea; 3 Department of Physics, University of Seoul, Seoul, 02504 Republic of Korea; 4 Energy Materials and Surface Sciences Unit, Okinawa Institute of Science and Technology Graduate University, Okinawa, 904-0495 Japan; 5 Graphene Research Institute and Department of Physics, Sejong University, Seoul, 05006 Republic of Korea; 6 Division of Materials Science, Nara Institute of Science and Technology, Nara, 630-0192 Japan; 7 Present Address: ARC Centre of Excellence in Exciton Science and Department of Chemical Engineering, Monash University, Clayton, VIC 3800 Australia

## Abstract

The valid strong THz absorption at 1.58 THz was probed in the organic-inorganic hybrid perovskite thin film, CH_3_NH_3_PbI_3_, fabricated by sequential vacuum evaporation method. In usual solution-based methods such as 2-step solution and antisolvent, we observed the relatively weak two main absorption peaks at 0.95 and 1.87 THz. The measured absorption spectrum is analyzed by density-functional theory calculations. The modes at 0.95 and 1.87 THz are assigned to the Pb-I vibrations of the inorganic components in the tetragonal phase. By contrast, the origin of the 1.58 THz absorption is due to the structural deformation of Pb-I bonding at the grain boundary incorporated with a CH_3_NH_2_ molecular defect.

## Introduction

Recently, organic-inorganic hybrid perovskite (OHP), ABX_3_ (A = Organic cation: CH_3_NH_3_^+^/NH_2_CH = NH_2_^+^, B = Metal cation: Pb/Sn, and X = Halide anion: Cl/Br/I) is a promising material for solar-cell, field-effect transistor, and light-emitting diode applications^1–7^. Because OHP material shows several advantageous properties such as wide light absorption range, low exciton binding energy, and high carrier mobility^4,8,9^. Also, the precursor materials for the fabrication of OHPs are cheap. Furthermore, OHP thin films can be fabricated using solution-based methods such as spin-casting and printing, which also corresponds to low-cost technologies^4^. Currently, the number of published works on OHPs are growing exponentially^10^. Even, nanoscience research using OHP material is just begun, and its single crystal formation with nano-scale size and physical properties such as bandgap engineering were reported^6,7,11–13^. New applications employing these OHP materials are expected to further expand due to the several unique physical properties still unexplored in these materials.

To understand the structural and fundamental properties of OHPs for a possibility of new application, THz-based measurement is a useful technique because the THz energy range (0.5~5 THz) is susceptible to the resonance of molecular vibration/rotation and lattice vibrations^4,14^. Recently, C. Quarti *et al*. reported the bending-stretching of the Pb–I bonds in CH_3_NH_3_PbI_3_ using Raman spectroscopy corroborated by density-functional theory simulations, that are diagnostic modes of the inorganic cage with the bands at 62 (1.86 THz) and 94 (2.82 THz) cm^–1^ ^15^. In this report, they conducted characterization on solution-prepared samples. An alternative method for the fabrication of OHPs reported in several works is based on vacuum-evaporation^16–18^. This might be a possibility to find a new physical property of the OHP thin films made by the different fabrication methods.

In this study, we performed THz time-domain spectroscopy (THz-TDS) to see the THz absorption property in OHP thin films made by 1) solution-processed (2-step and antisolvent methods) and 2) vacuum-evaporated (sequential vacuum evaporation: SVE) methods with CH_3_NH_3_PbI_3_ and CH_3_NH_3_SnI_3_^16,17,19^. Interestingly, the approximately 50% transmission of THz amplitude and 11000 cm^−1^ of absorption coefficient at 1.58 THz was found only from the CH_3_NH_3_PbI_3_ thin film fabricated by the vacuum-evaporated method, and this absorption is assumed to originate from a structural deformation incorporated with the molecular defect of CH_3_NH_2_ at the grain boundary. Finally, we suggest this can be a good possibility for THz-based applications using OHP thin film with cheap and easy-fabrication.

## Results

**Figure 1:**
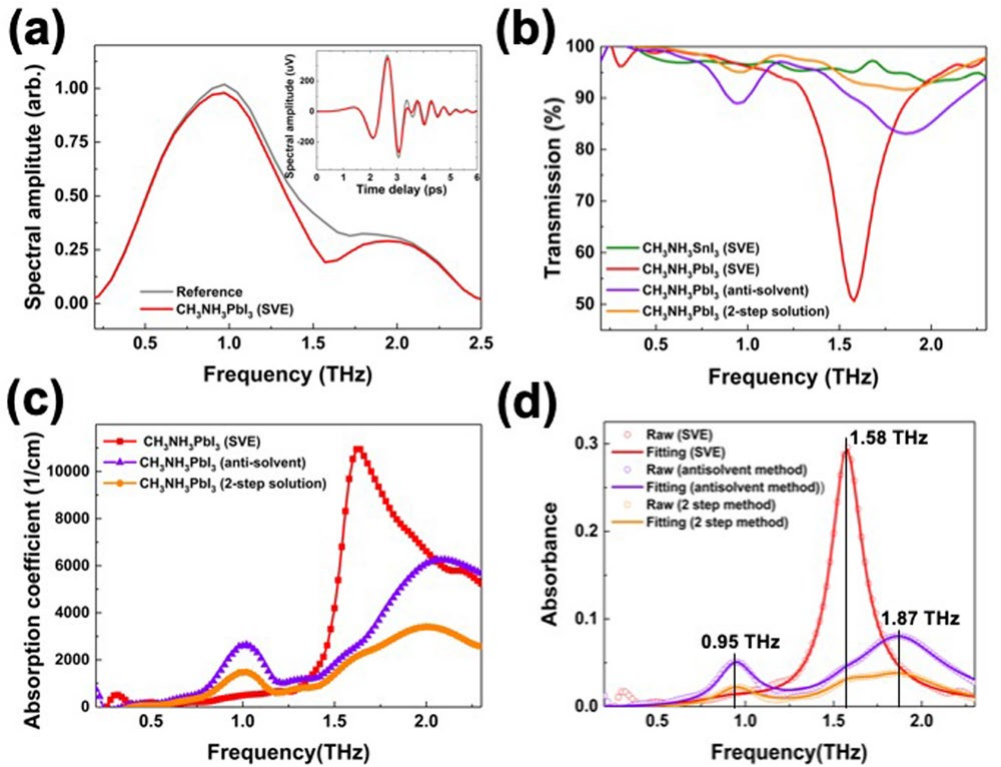
(**a**) Transmitted THz waveform through sapphire substrate reference (gray) with the CH_3_NH_3_PbI_3_ hybrid perovskites film fabricated by vacuum evaporation method (red) in frequency domain and time domain (inset). (**b**) THz transmission spectra with different fabrication methods. The transmission spectrum of the CH_3_NH_3_SnI_3_ thin film fabricated by the SVE method (green), the CH_3_NH_3_PbI_3_ thin film fabricated by SVE method (red), 2-step solution method (orange), and antisolvent method (purple). The 50% transmission at the 1.58 THz is observed in the SVE sample. (**c**) The absorption coefficients of the thin films fabricated by the SVE method (red), 2-step solution method (orange), and antisolvent method (purple). (**d**) The THz absorbance fittings using the three resonance frequencies with 0.95, 1.58, and 1.87 THz.

We demonstrate the THz-TDS on the CH_3_NH_3_PbI_3_ and CH_3_NH_3_SnI_3_ OHP thin film formed on Al_2_O_3_ substrate by solution-processed (2-step solution and antisolvent methods) and sequential vacuum-evaporated (SVE) methods^16–19^. In the transmission spectra (Fig. 1a,b), we found clear resonance features and significant absorption difference in each fabrication method. It is difficult to find representative absorption spectra in the CH_3_NH_3_SnI_3_ thin film which are seemed to be mixed with several absorption signals compared to the CH_3_NH_3_PbI_3_. These observations are correlated with the well-known material instability of Sn-based perovskite thin film^20^.

Two main phonon modes are observed at 0. 95 THz and 1.87 THz in the CH_3_NH_3_PbI_3_ thin films fabricated by the 2-step solution method and the antisolvent method^21^. The absorbance of the film made from the anti-solvent method is two times higher than that of the 2-step solution method. This result is consistent with the previous report^22^. The modes are assigned to the buckling of the Pb-I-Pb angles and Pb-I length vibrational mode^22^. On the other hand, the CH_3_NH_3_PbI_3_ thin film fabricated by SVE method shows one strong and well-resolved absorptance peak around 1.58 THz, which reaches almost 50% of transmission and 11000 cm^−1^ of absorption coefficient (Fig. 1c). These results show that the property and origin of the THz absorption in OHP thin films are different depending on the different fabrication methods. More importantly, the high absorption properties make OHP materials ideal for THz-based optoelectronic device applications employing versatile and low-cost fabrication methods^23^. To quantify the contribution of each vibrational modes, we also performed the THz absorption fittings using the dielectric function of collection of Lorentz Oscillators with the standard thin film approximation^24–26^ (Fig. 1d). The fitting parameters and results are shown in Table 1 and Fig. 1d, respectively. The THz absorption of CH_3_NH_3_PbI_3_ thin film fabricated by the antisolvent method clearly exhibits dominant peaks at 0.95 and 1.87 THz, whereas the THz absorption of the CH_3_NH_3_PbI_3_ thin film fabricated by the SVE method exhibits the main peak at 1.58 THz. In the case of the 2-step solution method, the THz absorption consists of three oscillators with resonance frequencies (oscillator strength) of 0.95 (2.5), 1.58 (2.5), and 1.87 (4.7) THz.

**Table 1:** The THz absorption fitting results using the dielectric function of collection of Lorentz Oscillators with the standard thin-film approximation^24–26^.

Fabrication method	ε_∞_	ω_0j_/2π [THz]	Ω_j_/2π [THz]	γ_j_/2π [THz]
SVE method (Vacuum)	3	0.95	2.70	0.50
		1.58	**10**.**50**	0.16
		1.87	1.00	0.50
2-step method (Solution)	3	0.95	2.49	0.26
		1.58	**2**.**45**	0.31
		1.87	4.68	0.54
Antisolvent method (Solution)	3	0.95	3.68	0.23
		1.57	**1**.**09**	0.15
		1.87	7.60	0.57

**Figure 2:**
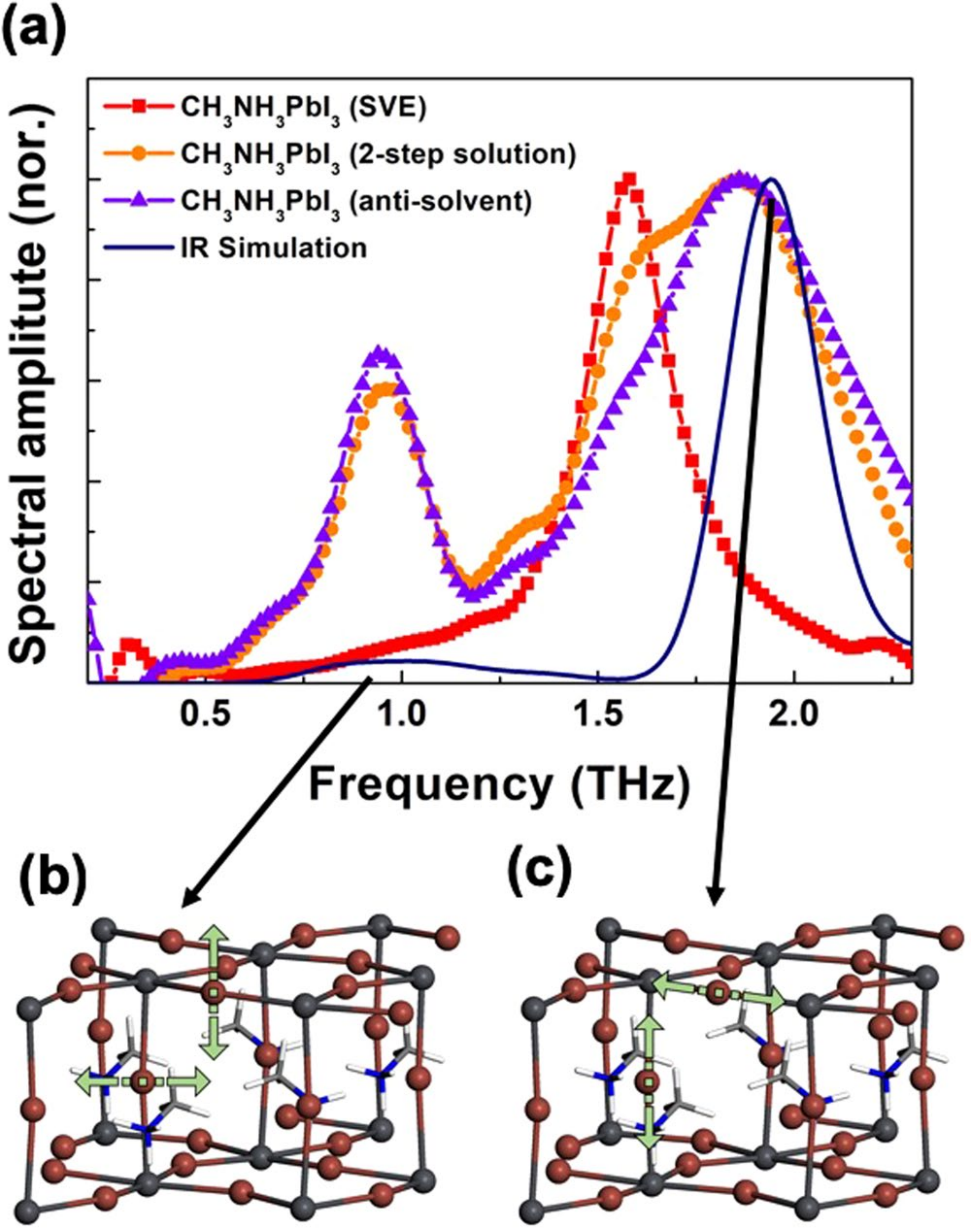
(**a**) The comparison with simulated IR spectra result. The theoretical vibration modes of (**b**) 0.95 and (**c**) 1.87 THz.

To understand the 0.95/1.87 THz and 1.58 THz absorptions from the solution-prepared and vacuum-evaporated methods, respectively, we performed infrared (IR) simulations using density-functional theory calculations of vibrational modes (Fig. 2). Firstly, we consider the tetragonal phase for the CH_3_HN_3_PbI_3_ system, which is the stable phase above 162 K. The IR simulation of CH_3_NH_3_PbI_3_ shows two main peaks at 0.95 and 1.87 THz in the low-frequency THz region (Fig. 2). These two modes are associated with the Pb-I vibrations of the inorganic components. At the vibration mode at 0.95 THz, the I atom between the two Pb atoms vibrates in the normal direction to the Pb-I bond (Fig. 2b). At the mode at 1.87 THz, the vibration of the I atom along the Pb-I bond is shown (Fig. 2c). This is in good agreement with our THz measurements for the CH_3_NH_3_PbI_3_ samples from the solution-based method and the previous theoretical results with vibration peaks near 1.0 and 2.0 THz. By contrast, the vibration mode at 1.58 THz obtained herein for the sample fabricated by the SVE method does not appear in the IR simulation of tetragonal CH_3_NH_3_PbI_3_. It means that the atomic structure of 1.58 THz is different from the 0.95/1.87 THz absorption. To understand this different THz absorption, we need to see more details.

**Figure 3:**
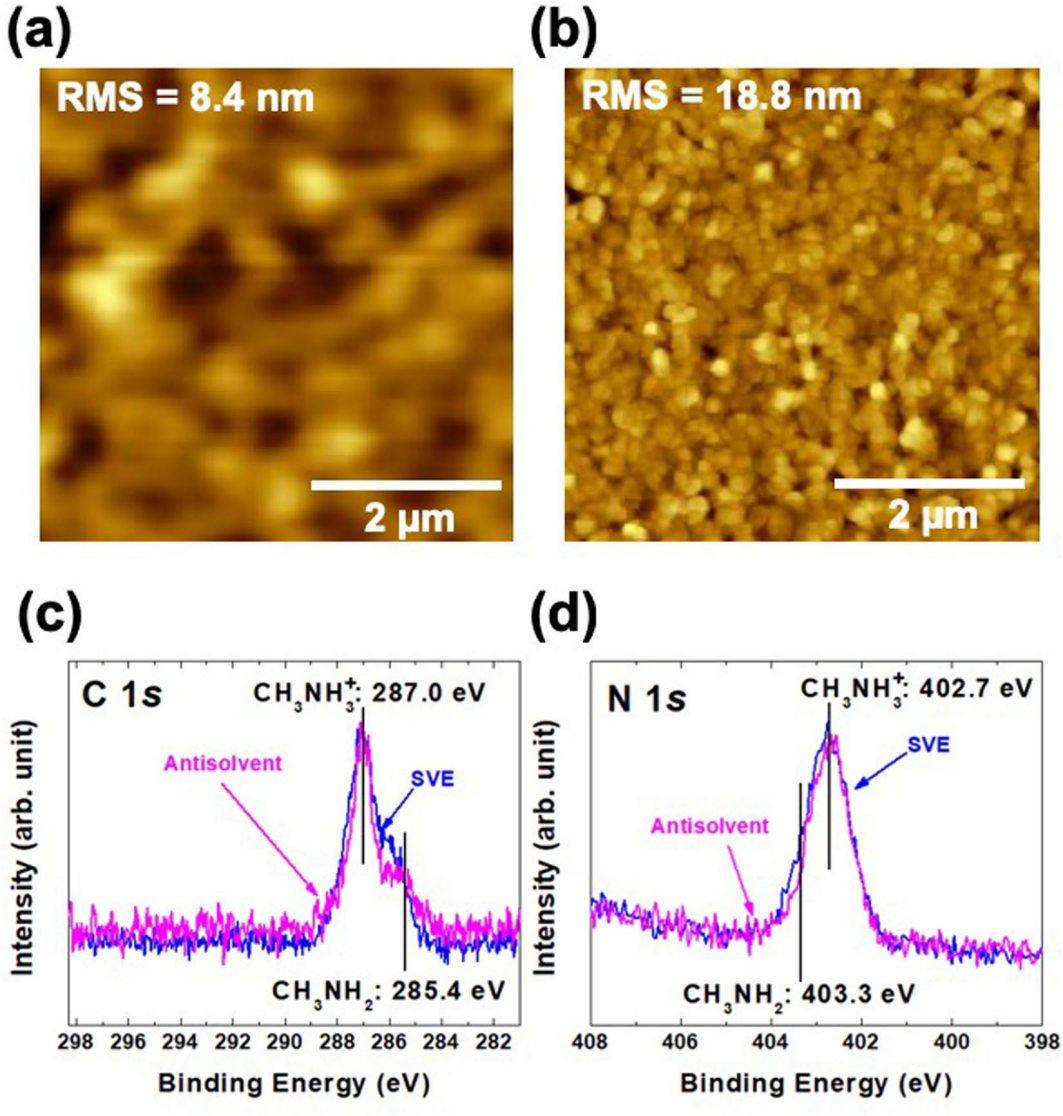
Surface morphology of (**a**) the antisolvent and (**b**) the SVE methods with 5×5 μm^2^. The surface roughness is 8.4 and 18.8 nm in the antisolvent and SVE methods, respectively. (**c**) C 1 *s* and (**d**) N 1 *s* core-level spectra. The CH_3_NH_2_ chemical states of the SVE sample are appeared with more intensity area.

**Figure 4:**
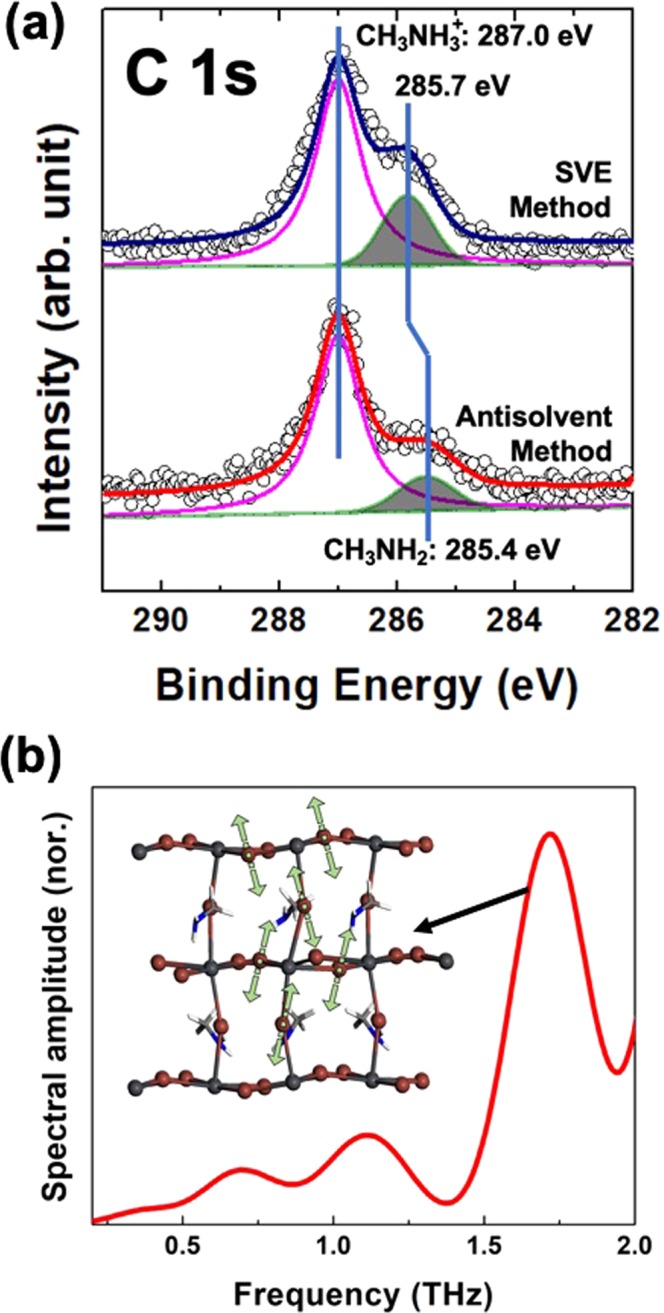
(**a**) The C 1 *s* core-level curve-fittings fabricated by the antisolvent and SVE methods. The CH_3_NH_2_ intensity ratio between the antisolvent and SVE methods is approximately 1:2. (**b**) Simulated IR spectra and vibrational mode of near 1.58 THz for CH_3_NH_2_PbI_3_.

The THz absorption is different depending on the fabrication method. In general, the main reports for the CH_3_NH_3_PbI_3_ thin film using the solution-prepared and vacuum-evaporated methods show a same atomic structure, chemical state, and optical bandgap. This means that the origin of 1.58 THz absorption is not originated from the typical CH_3_NH_3_PbI_3_ structure. In our previous report, we found the neutral molecular species, CH_3_NH_2_ at the grain boundary in the thin film fabricated by the 2-step solution-prepared method^18,19^. From this finding, the surface morphology and chemical state of the antisolvent and SVE samples were further investigated and compared using atomic force microscopy (AFM) and x-ray photoelectron spectroscopy (XPS) (Fig. 3). The surface morphologies from the antisolvent and SVE samples are observed to exhibit the different features (Fig. 4a,b). The size (density) of grain boundary of the SVE sample is smaller (denser) than that of the antisolvent sample. Moreover, the surface roughness is different as well. It means that the SVE sample has the larger density of grain boundary than the antisolvent sample. In the case of chemical state analysis, we found the different intensity ratio of CH_3_NH_2_ in the C 1 *s* core-level spectra (Fig. 3c). Consistently, the CH_3_NH_2_ intensity of N 1 *s* core-level in the SVE sample is larger than that in the antisolvent sample (Fig. 3d). We could not observe any different chemical state and intensity in the Pb 4 *f* and I 5*d* core-level spectra^18^. Only the different intensity area of CH_3_NH_2_ chemical states between the two samples are observed. In the case of contaminations such as water and oxygen, also, we could not observed^18^.

## Discussion

To have the quantitative analysis, we performed the XPS curve-fitting (Fig. 4a). We fitted C 1 *s* core-level spectra using Doniach-Sŭnjić curves convoluted with a Gaussian distribution of 0.5 eV FWHM^27^. Background due to inelastic scattering was subtracted by the Shirley (integral) method^28^. The different intensity area ratio between the antisolvent and SVE samples is found two times. The chemical shift with 0.3 eV in the SVE sample assumes to be a massive density of grain boundary made a more cation environment^29,30^. From these results, we assume the 1.58 THz absorption of the SVE sample is originated from the structural deformation in Pb-I bonding incorporated with the molecular defect, CH_3_NH_2_ neutral species^18,19^. And the SVE method can increase more density of CH_3_NH_2_ molecular defect than the solution-based methods. In order to find the vibration mode at 1.58 THz, we further consider the structural change of the inorganic cage associated with the incorporation of CH_3_NH_2_ instead of CH_3_NH_3_^+^ cation. In the geometry-optimized structure of CH_3_NH_2_PbI_3_, the Pb-I cage is significantly distorted (or deformed) and different from that of tetragonal CH_3_NH_3_PbI_3_ in the Pb-I bond distance and the I-Pb-I angle. Very interestingly, the IR simulation result shows a vibration mode near 1.58 THz (Fig. 4b). At the vibration mode at near 1.58 THz, the I atom between the Pb atoms vibrate in a complex way as shown in Fig. 4(b). This result shows that the vibration mode at 1.58 THz observed for the sample from the SVE method can be associated with the structural change of the Pb-I cage. This suggests that the incorporation of CH_3_NH_2_ instead of CH_3_NH_3_^+^ in CH_3_NH_3_PbI_3_ will be one of the possible origins for the structural deformation of the Pb-I cage associated with the appearance of the vibration peak shown in the sample prepared with the SVE method.

In summary, we have measured and unraveled the strong absorption at 1.58 THz in CH_3_NH_3_PbI_3_ thin film fabricated by the SVE method. The origin of the 1.58 THz absorption is due to the strong phonon resonance originated by the structural deformation incorporated with the molecular defect, CH_3_NH_2_ at the grain boundary. These findings are expected to allow a possibility of THz-based applications using OHP thin film fabricated by the SVE method. We believe that it requires to study more to understand a different property in a defect-incorporated hybrid perovskite structure.

## Methods

### The sample preparation

The Al_2_O_3_ substrates (made by Hi-Solar Co., Ltd.) are used for all sample preparations. The surface orientation is C-plane(0001) with the off-angle of 0.2° ± 0.05°. The thickness and roughness are 430 ± 25 μm and Ra ≤ 0.3 nm, respectively. 1) Solution-prepared methods – [2-step solution method]^19^ We performed spin-coating at 6000 rpm for 30 s on the sapphire substrates, previously heated at 70 °C. After the spin coating, the PbI_2_ layer was dried at 70 °C for 20 min. Then, a 20 mg/ml methylammonium iodide (MAI) solution in 2-propanol (IPA) was prepared and kept at 70 °C. PbI_2_ film was dipped in the MAI solution for 30 s with gentle shaking of the substrates. After dipping, substrates were rinsed in copious IPA and dried immediately by spinning them on a spin coater, after which they were annealed for 20 min on the hot plate at 70 °C. [Anti-solvent method]^31^ The CH_3_NH_3_PbI_3_ perovskite layer was deposited under ambient conditions using the anti-solvent method. The precursor solution was made by mixing 461 mg PbI_2_ (99.99%, TCI) and 159 mg methylammonium iodide (MAI, Dyesol) in 530 μL of DMF plus 73 μL of dimethylsulfoxide (DMSO). The solution was spin-coated at 2800 rpm for 25 s onto the substrate, and after 12 s spinning, 300 μL of diethyl ether were dripped onto the film. The transparent film was transferred to a N_2_-filled box and annealed at 60 °C for 10 min and then at 100 °C for 20 min. (See the SFigs 1 and 2 in the Supplementary Material). 2) Vacuum-evaporated samples - The CH_3_NH_3_PbI_3_ and CH_3_NH_3_SnI_3_ thin films were formed by the sequential evaporation method^16,18,20^, a 100 nm layer of Lead(II) iodide (PbI_2_, Alfa Aesar, 99.999%) and Tin(II) iodide (SnI_2_, Alfa Aesar, 99.999%) was deposited on a respective Al_2_O_3_ substrate followed by deposition of a 300 nm Methylammonium iodide (CH_3_NH_3_I, Dyesol) layer. And then the sample was loaded into the main chamber. The base pressure of chamber was maintained below 1.0 × 10^−6^ *Torr*. (To see the detailed characterization, see the reference of^18^).

### THz-TDS measurement

The THz transmission spectra were measured with a standard THz time-domain spectroscopy (THz-TDS) setup based on a femtosecond laser. (SFig. 3) A femtosecond Ti:sapphire laser (Mai Tai, Spectra-physics) which has 100 fs pulse width and 80 MHz repetition rate was divided into generation and detection parts. We used optical rectification generation with 1 mm ZnTe (110) crystal and electro-optical sampling detection with 2 mm ZnTe crystal. Two THz lens were mounted between the parabolic mirrors for focusing the THz beam the size of 2 mm diameter. We measure the THz time domain signal in the spectral range from 0.2 up to 2.5 THz. The transmitted THz frequency domain spectrum were acquired by the fast Fourier transforms (FFT) to each time domain THz pulse waveform.

### Theoretical calculations

For calculating IR and vibrational mode, we performed using density functional theory (DFT) as implemented in the code of the CASTEP. We used the On-the-fly norm-conserving pseudopotential generation in CASTEP (OTFG norm conserving) and the exchange-correlation functional of the spin-polarized Perdew-Burke-Ernzerhof expression revised for solids (PBEsol) in the generalized gradient approximation (GGA) in all cases. Electronic wave functions were expanded by plane waves with an energy cut-off of 990 eV. The geometry optimization of the tetragonal phase of CH_3_NH_3_PbI_3_ for bulk at room temperature were carried out until Hellmann-Feynman force acting on the atoms was smaller than 0.01 eV/Å without any symmetry constraint. For calculating infrared (IR) spectra and vibrational modes, we used density-functional theory (DFT) as implemented in the code of the VASP. We employed the projector augmented-wave method^32^ for the electron-ion interactions and the spin-polarized Perdew-Burke-Ernzerhof expression revised for solids (PBEsol) in the generalized gradient approximation (GGA) for the exchange and correlation interactions of electrons^33^. The electronic wave functions were expanded by plane waves with an energy cutoff of 450 eV. The geometry optimization was carried out without any symmetry constraint until the Hellmann-Feynman forces acting on the atoms were smaller than 0.01 eV/Å. For the bulk phase at room temperature, we used the tetragonal CH_3_NH_3_PbI_3_ structure, where the optimization of the cell volume and the atomic positions were performed^34^. And the CH_3_NH_2_PbI_3_ structure with the incorporation of CH_3_NH_2_ instead of CH_3_NH_3_^+^, we performed the optimization of the cell volume, the cell shape, and the atomic positions. To obtain the simulated IR spectra, we employed the finite difference method of the density-functional perturbation theory in VASP^35^ and the PHONOPY package^36^. The k-space integration was carried out with a 3 × 3 × 3 Monkhorst-Pack k-point mesh in the Brillouin zone of the supercell. The simulated IR spectra were broadened by convolution of a 0.1 THz Lorentzian to give the better comparison with the experimental observations.

### Thin film characterizations

All formed OHP thin films were characterized by atomic force microscopy (AFM) and x-ray photoelectron spectroscopy (XPS) using SPM-9700 (Shimadzu) and PHI5000 Versa ProbeII with a monochromated Al*K*α (ULVAC-PHI) to obtain the surface morphologies and chemical states, respectively.

## Supplementary information

Supplementary Information
