# Supplementary material for: Significant THz absorption in CH3NH2 molecular defect-incorporated organic-inorganic hybrid perovskite thin film
Source: Sci Rep. 2019 Apr 9;9:5811. doi: 10.1038/s41598-019-42359-8 (PMC6456617; doi:10.1038/s41598-019-42359-8)
Supplement: Supplementary file 1 — Supplementary Information [file 41598_2019_42359_MOESM1_ESM.pdf]

Supplemental Information

Title

Significant THz absorption in CH_3NH_2 molecular defect-incorporated organic-inorganic hybrid perovskite thin film

Authors

Inhee Maeng[1†], Young Mi Lee[2†], Jinwoo Park[3†], Sonia R. Raga[4‡], Chul Kang[1], Chul-Sik Kee[1], Byung Deok Yu[3], Suklyun Hong[5], Luis K. Ono[4], Yabing Qi[4], Min-Cherl Jung[6*] and Masakazu Nakamura[6]

Affiliations

[1]Advanced Photonics Research Institute, Gwangju Institute of Science and Technology, Gwangju, 61005, Republic of Korea

[2]Beamline department, Pohang Accelerator Laboratory, POSTECH, Pohang, 37673, Republic of Korea

[3]Department of Physics, University of Seoul, Seoul, 02504, Republic of Korea

[4]Energy Materials and Surface Sciences Unit, Okinawa Institute of Science and Technology Graduate University, Okinawa, 904-0495, Japan

[5]Graphene Research Institute and Department of Physics, Sejong University, Seoul, 05006, Republic of Korea

[6]Division of Materials Science, Nara Institute of Science and Technology, Nara, 630-0192, Japan

[*]Correspondence to: mcjung@ms.naist.jp

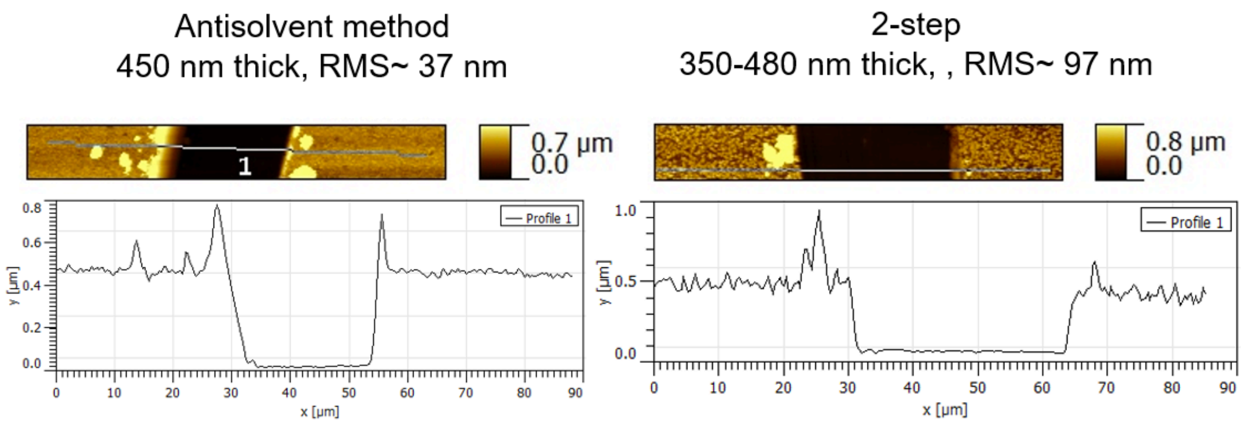

Fig. S1. Surface morphology of the solution-prepared samples confirmed by AFM.

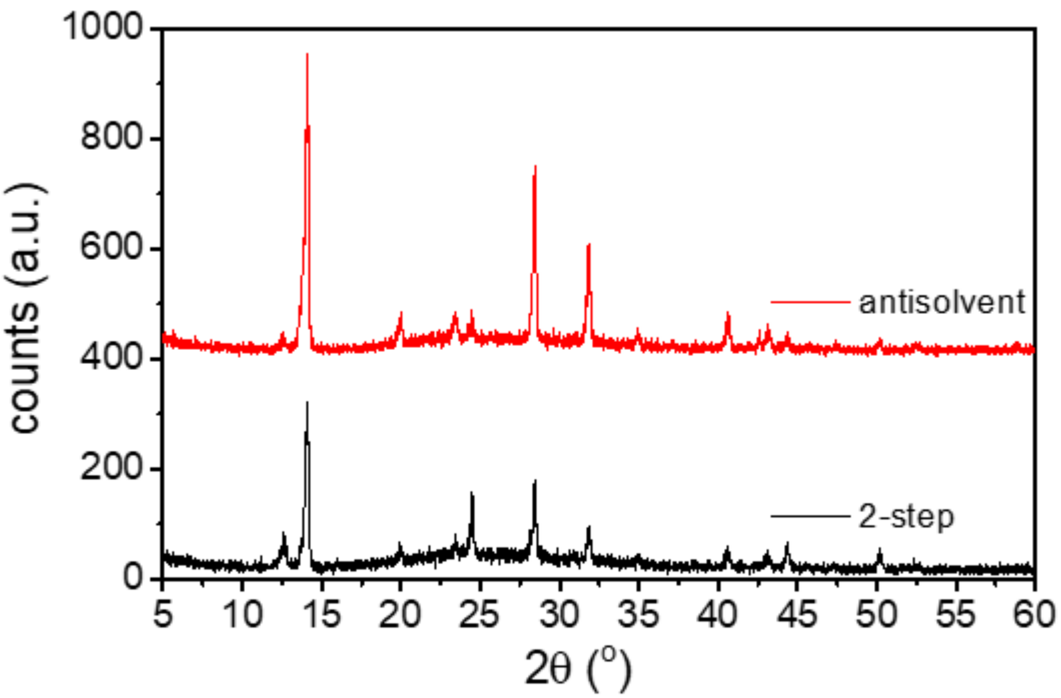

Fig. S2. XRD of the solution-prepared samples.

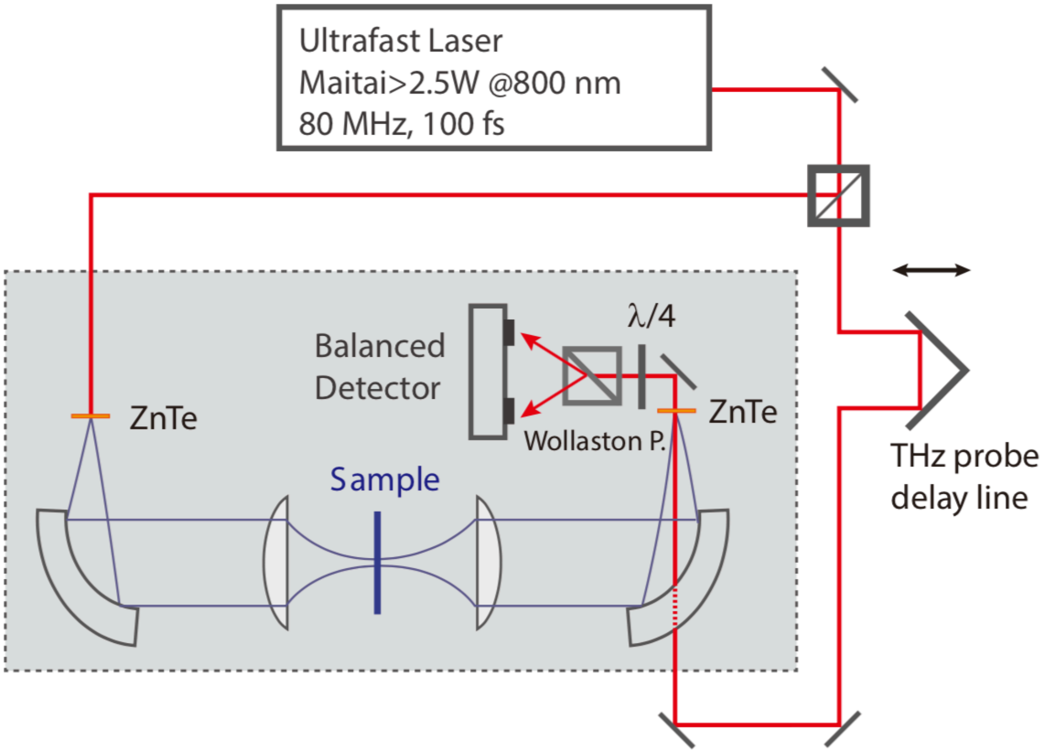

Fig. S3. A standard THz-TDS setup based on a femtosecond laser.
